# Is specific IgE antibody analysis feasible for the diagnosis of methylenediphenyl diisocyanate-induced occupational asthma?

**DOI:** 10.1007/s00420-012-0772-6

**Published:** 2012-04-28

**Authors:** Lygia Therese Budnik, Alexandra M. Preisser, Hjalmar Permentier, Xaver Baur

**Affiliations:** 1 Division of Occupational Toxicology and Immunology, Department of Occupational Medicine, Faculty of Medicine, Institute for Occupational and Maritime Medicine (ZfAM), University of Hamburg, Marckmannstrasse 129 b, Bld. 3, 20539 Hamburg, Germany; 2 Division of Clinical Occupational Medicine, Institute for Occupational and Maritime Medicine and Outpatient Clinic, University Medical Center, Hamburg Eppendorf, Germany; 3 Institute for Analytical Chemistry and Mass Spectrometry Center, University of Groningen, Groningen, The Netherlands; 4 Chair for Occupational Medicine, Medical Faculty, Institute for Occupational and Maritime Medicine, University of Hamburg, Hamburg Eppendorf, Germany

**Keywords:** Occupational asthma, Isocyanates, MDI, 4,4′-methylenediphenyl diisocyanate. (diphenylmethane-4,4′-diisocyanate), Specific IgE antibodies, Specific IgG antibodies, Immunological diagnosis, Albumin conjugates, Asthma diagnosis, Hypersensitivity pneumonitis, Isocyanate alveolitis

## Abstract

**Purpose:**

Early recognition improves the prognosis of isocyanate asthma. A major unanswered question is whether IgE-dependent mechanisms are of diagnostic value? Our objective was to appraise serological methods using various methylenediphenyl diisocyanate (MDI)-albumin conjugates and weigh up the data versus the outcome of standardized comprehensive clinical diagnostics to evaluate the viability of immunological analysis in supportive MDI-asthma diagnosis (OA_I_).

**Methods:**

Specific IgE (sIgE) and IgG (sIgG) binding was measured with fluorescence enzyme immunoassay in 43 study subjects (using conjugates prepared in-vapor, in-solution and commercial preparations). The differential clinical diagnosis included standardized measurement of pulmonary function, non-specific bronchial hyper-responsiveness, specific MDI-prick test (MDI-SPT) and specific inhalation challenge (MDI-SIC).

**Results:**

Detailed diagnostic scheme allows the differential OA_I_ and MDI-induced hypersensitivity pneumonitis (P_I_). The presumed OA_I_ diagnoses were confirmed in 84 % (45 % cases having demonstrable sIgE antibodies) with RR 5.7, *P* > 0.001, when OA_I_ diagnosis is correlated with MDI-SIC/MDI-SPT (RR 1.28 for MDI-SIC alone); sIgG antibodies were clinically relevant for P_I_ and not for the OA diagnosis. MDI-specific IgE data generated with commercial ImmunoCAP preparations show high correlation with our in-vapor generated MDI conjugates.

**Conclusions:**

Isocyanate-specific IgE antibodies are not always detectable but their presence is strongly predictive of OA_I_ and supportive for the diagnosis. MDI-SPT can be a valuable parameter differentiating OA_I_ and P_I_. We have confirmed and extended published data showing that isocyanate-albumin conjugates perform better in specific antibody assays when prepared with volatile phase formulations and would like to stress additionally the necessity for further refinements and standardization in clinical diagnostics procedures.

**Electronic supplementary material:**

The online version of this article (doi:10.1007/s00420-012-0772-6) contains supplementary material, which is available to authorized users.

## Introduction

Asthma is generally acknowledged as a critical endpoint after exposure to isocyanates (Malo and Chan-Yeung 2009; Maestrelli et al. 2009; Mapp et al. 1994), like 4,4′-methylenediphenyl diisocyanate (MDI) the most commonly used isocyanate. Individuals applying adhesives, paints, foams and other products (in construction, mining, agriculture, the shoe and automobile industries, or in orthopedic surgery) may be exposed to various volatile forms of MDI, accounting for about 60 % of global isocyanate consumption (World-Health-Organization 2000). The unequivocal diagnosis of occupational asthma after isocyanate exposure is difficult. A major unanswered question is whether IgE-dependent mechanisms are of diagnostic value or else are the available IgE tests inadequate for the purpose?

Reactive volatile isocyanates can access epithelial and mucosal compartments during inhalation and produce complexes with endogenous proteins, promoting their antigenicity in vivo. To elucidate the specific immune responses to such small-molecular-weight environmental chemicals in vitro, their conjugation with a relevant carrier host protein like albumin is needed. The structure of naturally occurring conjugates might influence their biological availability, half-life and antibody-binding capacity. Inflamed airways characteristic of asthma may result from an allergic reaction to these conjugates, with the generation of specific IgE antibodies. From the clinical perspective, isocyanate asthma is expected to be associated with the production of isocyanate-specific IgE antibodies detectable in immunological tests. However, the existing immunodiagnostic methods detect allergen-specific IgE antibodies mostly in a minority (20–50 %) of the patients suffering from isocyanate asthma (Wisnewski and Jones 2010). The reason is still unclear. Is it possible that isocyanate asthma has a different etiology from environmental asthma or that the pathophysiological mechanisms are, at least in part, IgE-independent? Or simply that the IgE antibodies remain undetected because the sampling time is too late after exposure? Or that the available formulations used in the conventional immunological tests are inappropriate? Fundamental to this dilemma is the appropriateness of the isocyanate-protein conjugates used in any antibody detection assays and their relevance to the immunogenic haptenic complexes (or newly formed antigenic determinants) formed during the pathophysiological conditions.

Various serological tests described in the literature use different isocyanate-albumin conjugates preparations to detect immunological responses. Published data obtained using HDI and TDI conjugates generated with their vapor phase suggest that there may be antigenic differences (in-vapor phase generated isocyanate-albumin conjugates versus in-solution phase) related to the biophysics of the conjugation reaction (Wisnewski et al. 2004; Wisnewski 2007). Furthermore, it was considered that vapor phase exposure would lead to limited isocyanate conjugation with albumin, which presumably reflects the pathophysiological conditions during occupational exposure to isocyanates (Wisnewski 2007). The importance of these findings should not be underestimated when combining the serological test results with well-defined clinical data for future diagnosis and preventive measures with asthma.

Unfortunately, relatively few publications provide all necessary individual diagnostic parameters with the relevant immunological data, precluding comparisons with clinical diagnosis (Wisnewski and Jones 2010). Frequently, either the data on antibody assays (in-house assay used in most studies) or the clinical information for the individual patients is lacking (i.e. only positive SIC is provided as indicator for isocyanate asthma), or it remains unclear how the dose response and the detection limits (LODs) were calculated (and if the available analytical standards were used), making useful comparisons between the clinical parameters and the serological data difficult. Since clinical examinations including lung function tests are often insufficient for reliable isocyanate asthma diagnosis and the available immunological tests identify only a proportion of the affected subjects, there is a need for improvement and standardization of existing diagnostic tests.

In an attempt to evaluate how the isocyanate conjugates influence the diagnostic sensitivity of the specific IgE immuno-fluorescence assay, we have adopted the existing methods to prepare MDI-HSA (human serum albumin) conjugates in-vapor and could observe a significant increase in the assay sensitivity as compared to the conjugates prepared in-solution. We have used this improved serological method to search for isocyanate-specific antibodies in serum of patients with well-characterized MDI-related asthma and control subjects. Standardized, comprehensive clinical diagnosis was performed. The major aim of the study was to investigate whether IgE-dependent mechanisms are of diagnostic value for patients with MDI asthma, to standardize the available antibody tests for variations in conjugate preparations (the art of the conjugation, the incubation time) and the clinical diagnosis for isocyanate asthma (vs. hypersensitivity pneumonitis). Data were collected and analyzed to determine the influence of the variations in conjugate preparation (in-solution, in-vapor and the available commercial preparation) on antibody binding and the relations with the comprehensive detailed clinical diagnosis. Detailed diagnostic criteria are provided for both isocyanate asthma and hypersensitivity pneumonitis).

## Methods

### Study population

**Fig. 1:**
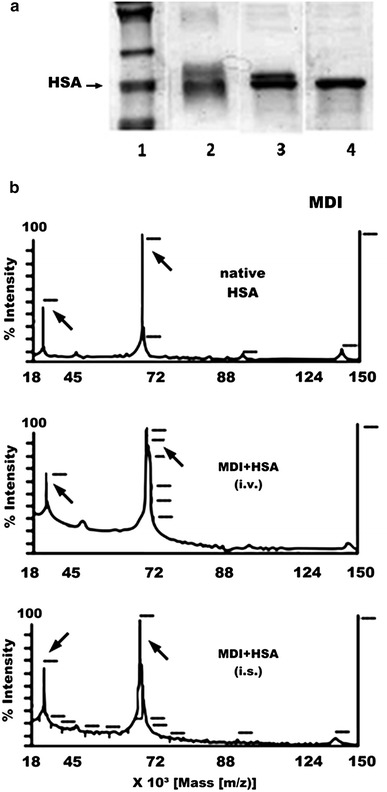
The 4,4′-MDI-HSA conjugates. **a** Protein gel analysis (polyacrylamide gel electrophoresis, SDS-PAGE) of the 4,4-MDI-HSA conjugates: lanes 1 = protein standards (*the arrows* show the positions for human albumin), 2 = 4,4′-MDI conjugate prepared in-solution (i.s.), 3 = 4,4′-MDI conjugate prepared in-vapor (i.v.), 4 = native HSA (no conjugate). **b** Mass spectrometry analysis (MALDI-TOF-MS) of 4,4′-MDI-HSA conjugates prepared using in-solution (i.s.) and in-vapor (i.v.) methods

We analyzed 43 persons, which include all patients with occupational exposure to MDI and presumed isocyanate asthma who were referred to our outpatient clinic by general practitioners in the last 5 years (*n* = 12). Three additional control groups were also studied: 6 asymptomatic industrial workers currently exposed to ~5 ppb MDI investigated in the workplace, 12 patients with occupational baker’s asthma, not exposed to isocyanates, and 13 unexposed healthy control subjects. The median value for the demographic, clinical and functional characteristics of the symptomatic patients and the controls were as follows: patient age 43 year (27–67), controls 46 year (28–61), in the patient group 91 % were men and in the control group 61 %; the total IgE values for the patient group were 102 kU/L IgE (2–1669), for the control group 92 kU/L (7–893); the median FEV1/FVC ratio in the MDI-exposed patient group was 0.79. Smoking status: 33 % of the patients were smokers, 8 % non-smokers and 58 % ex-smokers; in the control group: 11 % were smokers, 64 % non-smokers and 14 % ex-smokers. The patients and controls filled in questionnaires regarding their workplaces, working conditions, exposure, respiratory symptoms and smoking habits (the smoking status was confirmed with cotinine measurements); The patients underwent an extensive asthma examination (see Tables 1, 2; Fig. 1 for details). None of the isocyanate asthma patients (and controls) was under medication at the time of the study. The clinical, demographic and functional characteristics of the individual subjects are delineated in the results, as appropriate. The study was approved by the Institutional Ethics Review Board, (IRB0003648, Hamburg, Germany).

**Table 1:** Clinical diagnosis of symptomatic patients with MDI exposure history

Clinical diagnosis criteria of patients with presumed asthma and MDI exposure history
Individual diagnosis criteria follow the ERS guidelines (see text for details)
Differential diagnosis and exclusion (COPD, pulmonary embolism, congestive heart failure, pulmonary infiltration with eosinophils, vocal cord dysfunction, aspiration pneumonia, anti-inflammatory medication, mechanical obstruction, tumor)
Conditions, see isocyanate asthma diagnostic flow chart
Key indicators: obstructive ventilation pattern, recurrent wheeze, difficulty in breathing, chest tightness, poor respiratory effort
Typical exposure-related asthmatic symptoms, asthmatic symptoms occur or worsen at work; occupational MDI-related exposure history, isocyanate exposure assessment, presence of workplace-related bronchial hyperresponsiveness after exposure to MDI, positive MDI-SIC result, positive MDI-SPT result
Additional criteria considered were
Lung function: FEV1 below lower limit of normal, presence of non-specific bronchial hyperresponsiveness (NSBHR)
Presence of MDI-specific IgE antibodies

**Table 2:** Diagnosis of MDI hypersensitivity pneumonitis with evaluation points for isocyanate alveolitis

Routine diagnostic procedure for isocyanate alveolitis (with evaluation points)
Case history: typical exposure-related symptoms, see above (3 points)
Auscultation, crackles on lower lung fields (2 points)
Serological test: IgG antibodies to MDI-HSA conjugates (1 point)
Lung function tests: restrictive ventilation pattern, impaired diffusion capacity (2 points)
Chest X-ray: lung infiltrates ground or glass pattern (2 points)
Facultative diagnostic parameters
BAL: lymphocytosis, CD4/CD8 < 1 (3 points)
Serial lung functions testing demonstrating work-related FVC decline of at least 20 % points)
Specific inhalative challenge test: systemic inflammatory response plus restrictive ventilation response and/or impairment of diffusion capacity of at least 15 % (4 points)
Systemic inflammatory response (fever, leukocytosis) (3 points)
Positive diagnosis: when at least 10 points

### Pulmonary function test

FVC (forced vital capacity) and FEV1 (forced expiratory volume in 1 s) were measured according to ERS/ATS recommendations applying reference values from (Brandli et al. 1996, 2000).

### NSBHR (non-specific bronchial hyper-responsiveness)

The protocol for NSBHR testing has been described elsewhere (Baur et al. 1998). Briefly, the inhalation challenge involved serial measurements of FEV1 with progressively increasing doses of methacholine (up to 0.4 mg as measured at the mouthpiece). A 20 % fall of FEV1 elicited by ≤0.3 mg of methacholine (PC_20_ < 0.35) indicates NSBHR (Baur et al. 1998; Jayet et al. 2005).

### SPT (skin-prick testing)

SPT was performed with 20 common allergens following a protocol described earlier (Budnik et al. 2011; Baur et al. 1994). For specific MDI-SPT, sterilized, purified HSA-MDI conjugates were prepared: the 96 % sterile albumin solution (for human use from CSL Behring, Germany) was mixed (in solution) with sterile liquid monomeric MDI (Bayer, Germany) until a final concentration of 1 mg/mL MDI was achieved.

The allergens were gently pricked onto the skin surface of the volar side of the forearm. Wheal and flare reactions were read 20 min later (a test result was regarded as positive when a wheal of at least 3 mm in diameter appeared, with a surrounding flare, which was larger than the solvent, that is, negative control). The solvent alone (0.9 % sodium chloride) and histamine (0.01 mg/mL) were tested in parallel as negative and positive controls.

### SIC (specific inhalation challenge)

The SIC method performed in exposure chamber (0.5–5.5 ppb for 120 min) described elsewhere (Baur et al. 1994; Budnik et al. 2011). FEV1 was measured before and every 10 min for the 1st h, then hourly for 7 h. The SIC result was considered positive when the fall in FEV1 was at least 20 %.

### Clinical diagnosis of patients with MDI exposure history

The individual asthma diagnosis for each patient followed the ERS/ATS guidelines (Anees et al. 2011; Moore et al. 2010; Vandenplas et al. 2011; Tarlo et al. 2008; Baur et al. 1998) as described in detail below. See Table 1, for the schematic diagnostic criteria and supplementary Fig. 1 for diagnostic flow chart of the MDI-asthma diagnosis (see Figure 1 in supplementary material).

### Facultative diagnostic testing

In case of uncertainness due to clear-cut work-related symptoms (e.g. associated with the absence of NSBHR), additional spirometry monitoring and/or additional specific inhalative challenge tests were performed (supplementary Fig. 1).

### Diagnosis of MDI hypersensitivity pneumonitis (MDI alveolitis)

Diagnosis of MDI hypersensitivity pneumonitis has been described in detail elsewhere (Baur et al. 1992, 2001; Merget et al. 2002). Prerequisites of acute or subacute MDI hypersensitivity pneumonitis are the following:

- Occupational/environmental history: MDI exposure.
- Respiratory as well as systemic symptoms after a lag period of 3–12 h: fever, shivering, malaise, cough and shortness of breath.

Diagnostic scheme in case of presumed MDI hypersensitivity pneumonitis is shown in the Table 2.

### Exposure assessment

Exposure assessment was performed using the MDA-SPM toxic gas monitor (Honeywell Analytics, Glinde, Germany) and was confirmed by biomonitoring (Budnik et al. 2011). If workplace measurement was not possible, the assessment of exposure was based on occupational case history, detailed reconstruction of the working conditions, data provided by industrial hygienists as well as information provided by the employees.

### Preparation of various MDI-HSA conjugates and immunological analysis

The preparation of MDI-HSA conjugates in-vapor and in-solution is a modification of previously published methods (Wisnewski et al. 2004; Sepai et al. 1995; Kumar et al. 2009; Baur 1983). The in-vapor method is based on a specially constructed 2 chamber-system used to fumigate the human albumin (99 % pure, globulin free, Sigma, Germany) solution with vaporized 4,4′ MDI (analytical standard, Riedel-de-Häen, Sigma, Germany). Individual conjugates, were coupled with biotin and used for the fluorescence enzyme immune assay detection method (semi-automatic ImmunoCAP100, Phadia, Freiburg, Germany). Serum-specific IgE is expressed in kilo unit per liter (kU/L) correlated with the WHO reference of human serum IgE (1 kU = 2.4 ng/mL). A seven-point dose–response calibration was performed for each IgE and IgG measurement. For ImmunoCAP-specific IgE, the limit of detection (LOD) of 0.02 kU/L for IgE and 0.2 mg/L for IgG and the limit of calibration of 100 kU/L for Commercial ImmunoCAP conjugates (K76, Phadia) used in routine clinical laboratories were applied in parallel with similar analytical procedures (for the calibration curves and control sera). For validation of the assays, the following controls were included: pooled positive and negative patient/control sera, analytical standards (also used as set points for quality control), HSA solution and biotin control samples. The measured day to day precision was <12 % RSD. The assay validation was performed according to the good laboratory practice. Separate studies with HSA solution showed that IgE values above 0.02 kU/L and IgG values above 3 mg/L can be considered as specific (above means +2 RSD or 10 % analytical variation). The variability between the in-vapor method and the commercial assay method was: 0.5–20 % (for lower and upper edge of failure) for the IgE values. For the IgG data, however, the values collected with commercial CAPs were continuously 5–35 % higher in all tested subjects.

Total IgE antibodies were determined using respective commercial Uni-CAP from Phadia.

### Detection of MDI-bound to HSA

The protein concentration of each test conjugate was determined by the method of Bradford (BioRad, Germany). The concentrations were adjusted by dilution or limited evaporation on a speed-vac system. The conjugates were subjected to SDS-PAGE using a 9 % separation gel. The amount of MDI-bound to HSA was calculated from the intact protein shift using MALDI-TOF-MS (using CHCA-matrix) and compared with non-conjugated HSA.

### LC-MS/MS measurements

Purified HSA was incubated with MDI and analyzed by MALDI-TOF mass spectrometry (Applied Biosystems, the Netherlands) to determine the mass shift of the intact protein. Additionally, the reacted HSA was digested with trypsin (without any further treatments, such as disulfide bond reduction). The digested mixtures were analyzed by liquid chromatography (LC)-mass spectrometry (MS) (Applied Biosystems, the Netherlands), and modified peptides were scanned using neutral loss and precursor ion scans. Interesting ions were analyzed again with product ion scans to identify them from their fragmentation spectra (data not shown).

### Data analysis

Immunological data are expressed as mean value. Each analysis was repeated at least twice with three independent preparations (except for the assay validation). For correlations between diagnosis probability estimates and the specific immunoglobulin binding, the relative prevalence ratios (RR) were calculated from the contingency tables using a logistic model. Two-sample *t* tests were applied to calculate the distribution of the difference. To calculate correlations, the Person’s correlation test was applied. When the clinical data were combined in union (i.e. NSBHR, MDI-SIC, MDI-SPT, sIgE), the results of tests in combination had to be positive; if any result was negative, the combination was considered negative. When clinical lung function parameters were evaluated, the percent of the predicted lung function values was calculated, applying the reference values of Brändli et al. (see “Methods”). For the comparison of the binding data between the sera for variously responding patients, the data for each individual patient were transformed into a percentage of maximal binding (i.e. if the maximum binding value was 10 kU/L, the 10 would be 100 % and other data points were given as a percentage of this value; if the maximum value was 70 kU/L, then 70 would be 100 %, thus allowing to compare high and low responds within one plot). The patient sera were measured first individually, and then the samples were pooled as follows: all IgE-positives (median, 26 kU/L) gave one pool, all IgG-positives (median, 13 mg/L) gave another, and two control pools (healthy group and baker’ asthma patients) were the third and the last group. When data point for only one conjugate is shown, the following conditions were chosen: in-vapor conjugates were used in AmBic buffer, 60 min-incubation (if not otherwise specified).

To test individual conjugates and to validate the assay, a pool serum from isocyanate asthmatics was used. All immunological methods were validated routinely with control serum samples and additional standard set points (two analytic standards, one low and one high concentration were used as set points). Two-sample *t* tests were applied to calculate the distribution of the difference. The data analyses were performed with GraphPAD Prism Software (GraphPad Software Inc, San Diego, CA).

## Results

### The antibody binding was higher in MDI-albumin conjugates prepared with volatile MDI as compared to the insoluble form, showing concomitant higher rates of the MDI incorporation on the other hand

**Fig. 2:**
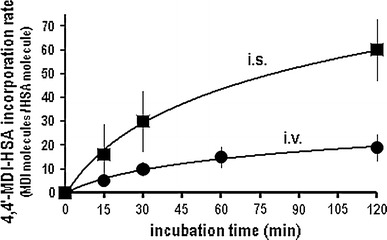
The preparation of the MDI-HSA conjugates influences the 4,4′-MDI incorporation rates into HSA. The MDI-HSA preparations in volatile form show lower isocyanate incorporation rates when compared with conjugates prepared in-solution. MDI incorporation rate for various 4,4′-MDI conjugate prepared in-solution (i.s., *filled square*) and in-vapor (i.v., f*illed circle*) was calculated as predicted number of MDI molecules per HSA molecule

We have tested exhaustively isocyanate-albumin conjugates with 4,4′-diphenylmethane diisocyanates (MDI), generated in-solution (i.s.) and in-vapor (i.v.) using different buffer systems (i.e. PBS and AmBic buffers) and incubation times. Compared with unreacted HSA, the protein gel analysis revealed a distinct molecular weight shift for the in-vapor prepared MDI conjugates, whereas the in-solution prepared conjugates produced a broad smear of additional high-molecular-weight bands (presumably representing heterogenous protein complexes cross-linked by di-isocyanates) (Fig. 1a). Figures 1b and 2 depict the comparison between the 4,4′-MDI-HSA protein conjugates in terms of the isocyanate incorporation rate for protein adducts prepared using formulations with liquid; i.s. and volatile, i.v. MDI.

**Fig. 3:**
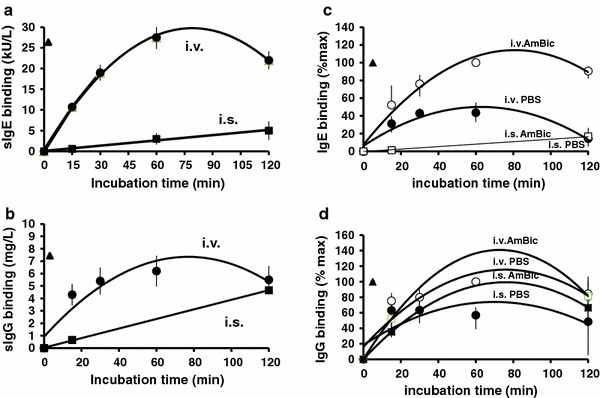
The influence of the MDI-HSA conjugate preparation conditions on antibody-binding capacities in fluorescent enzyme immunoassay. Specific IgE(**a**/**c**) and IgG(**b**/**d**) binding in patients’ sera. **a/b** 4,4′-MDI-HSA conjugates were prepared in-vapor (i.v.) and in-solution (i.s.) using PBS or AmBic. Specific IgE and IgG binding was tested using serum from MDI-exposed patients using the validated ImmunoCAP analysis. Data show different conjugate preparations (repeated twice, *n* = 3) tested with pooled patient sera. **c/d** Sera for each individual patient were measured and the binding data normalized against maximal binding (to allow comparisons between individual patients showing different maximal binding rates). Mean values (with min./max *error bars*, *n* = 12) are shown and calculated for specific IgE and IgG binding. Trend lines were generated using individual data points for various incubation times and buffers as indicated. The x-axis shows the incubation time during conjugate preparation. in-solution, i.s. = squares (*filled square*, *open square*) in-vapor, i.v. = circles (*filled circle*, *open circle*); commercial conjugate preparations = triangles (filled triangle); Phadia, PBS = solid symbols (*filled square*, *filled circle*); AmBic = empty symbols (*open square*, *open circle*)

When using soluble isocyanate, the MDI incorporation rates into albumin were higher than with the volatile form (Fig. 2). Conversely, conjugates prepared using the volatile MDI form (i.v.) showed much higher specific IgE and IgG antibody-binding capacities than did the conjugates prepared in the liquid form (i.s.) (Fig. 3a, b). The binding capacity (specific IgE and IgG binding) of the newly formed MDI-albumin conjugates was assessed using sera from patients with MDI-isocyanate asthma and control subjects (patients with non-isocyanate asthma, no isocyanate exposure and healthy control subjects).

In parallel, comprehensive differential clinical diagnosis schema (including specific inhalation challenges with MDI) was established (Tables 1, 2; supplementary Fig. 1) and was applied to the tested subjects. The patient data are given in the methods section (see also Tables 3, 4). Marked differences in binding capacities were observed for the various conjugate preparations, buffers, and incubation time periods (from 0 to 120 min) for both IgE and IgG (Fig. 3). The specific IgE binding to MDI-HSA was better for conjugates prepared in AmBic than in PBS (Fig. 3a, c). The choice of buffer also had some effect on the amount of specific IgG binding (see Fig. 3c, d).

**Table 3:** Demographic, clinical and functional characteristics of the symptomatic patients with MDI exposure history and presumed isocyanate asthma

Patient no. #	Demographic data			MDI exposure. (lag time) year	Art of exposure to MDI (job description)	Immunological status		Duration of resp. sympt (year)	Lung function			SPT MDI-HSA	MDI-SIC	MDI-HSA-specific antibodies		Final clinical diagnosis	
	Sex	Age	Smoking status			SPT comm. allerg.	Total IgE kU/L		FVC % pred	FEV1 % pred	NS-BHR			MDI-sIgE kU/L	MDI-sIgG mg/L		
Group A: MDI-exposed patients referred to our clinic with presumed isocyanate asthma diagnosis																	
1	M	29	Yes	5.5 (1)	MDI-PUR glue heated; harder, binder	Pos.	279	4	86	76	Pos.	Pos.	Pos.	13.3	<3	OA_I_	
2	M	63	Yes	14 (0.8)	MDI-PUR synthesis	Pos.	1669	12	97	69	Pos.	Pos.	Pos.	50.4	7.3	OA_I_	
3	M	36	Ex	3 (1)	MDI-PUR manufacture; MDI-lack bystander	Neg.	427	1	90	60	Pos.	Pos.	Pos.	4.8	9.6	OA_I_	
4	M	34	Ex	14 (0.7)	MDI-PUR glue heated, MDI cont. coatings	Pos.	226	8	97	94	Pos.	Pos.	Pos.	3.3	<3	OA_I_	
5	M	57	Ex	4 (0)	MDI-PUR foam manufacture	Pos.	61	3.4	74	78	Pos.	Pos.	Pos.	<0.02	<3	OA_I_	C_I_
6	M	54	Ex	5 (0)	MDI cont. production of elastomers	Neg.	102	4	85	58	Neg.	Neg.	Pos.	<0.02	74.0		P_I_
7	M	35	Ex	0.4 (0)	MDI-PUR cont. plastic manufacture	Pos.	51	0.4	81	69	Pos.	Neg.	Pos.	<0.02	4.9	OA_I_	
8	M	47	No	11.5 (0)	MDI-PUR electrical potting,	Neg.	15	10.5	79	68	Pos.	Neg.	Pos.	<0.02	20.2		P_I_
9	M	49	Yes	11 (0)	MDI-PUR manufacture of. hard plastic parts	Neg.	8	2.5	85	62	Neg.	Neg.	Pos.	<0.02	3.3	OA_I_	
10	F	43	Yes	0.3 (0)	MDI-PUR-durable elastomeric wheels,-foam	Neg.	108	0.1	100	57	Pos.	Neg.	Pos.	<0.02	14.8	A_1_	P_I_
11	M	49	Ex	13 (0.8)	MDI glue, heated, plastic, wood panels	Neg.	12	6	79	72	Neg.	Neg.	Neg.	<0.02	3.6		P_1_
12	M	43	Ex	2 (0.2)	MDI-PUR powder, acryl lack parts	Neg.	2	1.5	81	73	Pos.	Neg.	Neg.	<0.02	3.7	A_1_	

M, Male; F, Female; comm. allerg., common allergens; MDI exp. duration of work-related exposure to MDI; lag time, lag time since last exposure; resp. sympt, duration of reported respiratory symptoms; FVC, forced vital capacity; FEV1, forced expiratory volume in 1 s; NSBHR, non-specific bronchial hyper-responsiveness; MDI-SIC, MDI-specific inhalation challenge; sIgE, MDI-specific IgE; sIgG, MDI-specific IgG. OA_I_, occupational MDI asthma; P_I_, MDI-induced hypersensitivity pneumonitis; D_I_, dermatitis, due to MDI; C_I_, conjunctivitis due to MDI; RC_I_, rhino-conjunctivities, due to MDI; A_1_, work-aggravated isocyanate asthma (aggravated by MDI exposure) at the time of blood sampling; P_1_, early stage of hypersensitivity pneumonitis due to MDI (isocyanate alveolitis, that is, mild clinical symptoms and non-significant changes in lung function occurred in the challenge test); n.d., not determined; H, healthy

**Table 4:** Demographic, clinical and functional characteristics of MDI-exposed asymptomatic industrial workers

Patient no. #	Demographic data			MDI exposure. year (*PPE)	Biomonitoring MDA values (at the time of sampling) Air monitoring. median value 5 ppb	Immunological status		Reported duration of resp. sympt (year).	Lung function			SPT MDI-HSA	MDI-SIC	MDI-HSA-specific antibodies		Final clinical diagnosis
	Sex	Age	Smo-king status			SPT comm. allerg.	Total IgE kU/L		FVC % pred	FEV1 % pred.	NS-BHR			MDI-sIgE kU/L	MDI-sIgG mg/L	
Group B: Workplace field controls; workers currently exposed to MDI																
1	M	38	Yes	11.3	0.16 μg MDA/g Creatinine	Neg.	39.3	–	98	84	n.d.	n.d.	n.d.	<0.02	<3	RC_I_
2	M	43	Yes	10.1	0.90 μg MDA/g Creatinine.	Neg.	42.9	–	102	98	n.d.	n.d.	n.d.	<0.02	<3	RC_I_
3	M	33	Yes	8.2 (*)	0.30 μg MDA/g Creatinine	Neg.	97.3	–	104	84	n.d.	n.d.	n.d.	0.25	3.5	H
4	M	33	No	7.7	0.32 μg MDA/g Creatinine	Neg.	37.7	–	97	88	n.d.	n.d.	n.d.	<0.02	<3	C_I_
5	M	32	Yes	5.5	0.20 μg MDA/g Creatinine	Neg.	13.3	–	109	91	n.d.	n.d.	n.d.	<0.02	<3	C_I_
6	M	25	No	2.1	0.22 μg MDA/g Creatinine	Pos.	28.6	–	96	92	n.d.	n.d.	n.d.	<0.02	<3	RC_I_D_I_

The six industrial workers involved in the production of MDI cont. coatings reported to have no respiratory symptoms (questioner) before being enrolled for the analysis. 5 showed RC/C symptoms after the work week, only one worker hat no measurable symptoms. Only one worker was wearing the personal protective mask (PPE) during the whole work shift M, Male; F, Female; comm. allerg., common allergens; MDI exp. duration of work-related exposure to MDI; lag time, lag time since last exposure; resp. sympt, duration of reported respiratory symptoms; FVC, forced vital capacity; FEV1, forced expiratory volume in 1 s; NSBHR, non-specific bronchial hyper-responsiveness; MDI-SIC, MDI-specific inhalation challenge; sIgE, MDI-specific IgE; sIgG, MDI-specific IgG. OA_I_, occupational MDI asthma; P_I_, MDI-induced hypersensitivity pneumonitis; D_I_, dermatitis, due to MDI; C_I_, conjunctivitis due to MDI; RC_I_, rhino-conjunctivities, due to MDI; n.d. not determined; H, healthy

There was a linear correlation between both the IgE and IgG values collected with either our fluorescence immunoassay using in-vapor conjugates and the commercially available ImmunoCAPs (Phadia) analysis with *r* = 1.00 and *r* = 0.79 (for IgE and IgG, respectively). Because of this high correlation, one can presume that these commercial conjugates were made in-vapor. All positive and negative antibody values in reactive and non-reactive subjects correlated between the two CAP systems within a permissive assay variability of 0.5–20 % for the absolute sIgE values. For the IgG data, however, the values collected with commercial CAPs were up to 35 % higher (resulting in false-positive values in lower range).

### Clinical diagnosis and antibody analysis

To address the question on the diagnostic feasibility of antibody testing for the isocyanate asthma diagnosis, we have analyzed the data from patients with presumed asthma diagnosis referred to our polyclinic from general practitioners. In order to evaluate the results of the immunological tests against the clinical diagnosis, two steps are needed in each case: a comprehensive diagnostic approach and validated serological test. Our 12 patients underwent specific inhalation challenges with MDI (none of the control subjects did approve for either SIC or MDI-prick tests). Their atopy status, skin-prick test results, serial lung function testing, demographic data and clinical diagnosis are given in Tables 3, 4. Four subjects showed positive specific IgE reaction (3.3–50.4 kU/L of sMDI-IgE) and 10 had specific IgG antibodies: (3.5–74 mg/L sMDI-IgG); 4 MDI-asthma patients showed low values of sIgG (3.3–9.6 mg/L sIgG; 0.3–6.6 mg/L higher than the unspecific settled value of 3 mg/L), whereas the 4 hypersensitivity pneumonitis patients had mostly higher sIgG values (up to 74 mg/L).

**Fig. 4:**
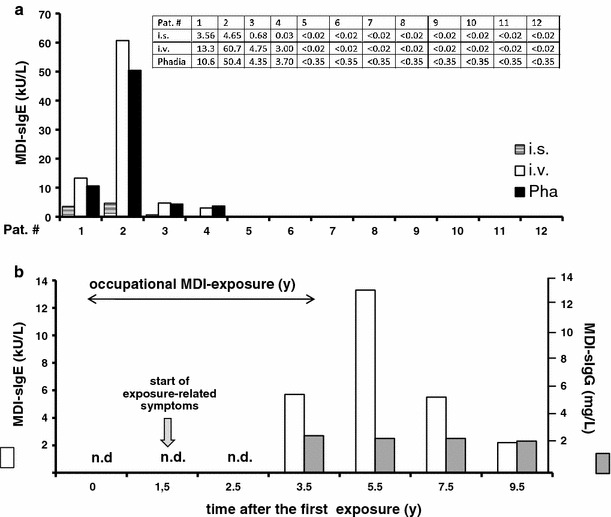
Specific IgE antibody level may persist for several exposure-free years. **a** Serum IgE antibody levels for all patients with presumed MDI-asthma (see Tables 3, 4 for patient details) measured with fluorescence enzyme immune assay using MDI-HSA conjugates prepared either, in-solution (i.s., hatched columns), in-vapor (i.v., solid white columns), or commercial (Phadia, Pha, black column) conjugates (see methods in Appendix l for more details). **b** Serum sIgE antibody levels for one MDI-asthma patient (pat#1, Tables 3, 4) in a longitudinal study during MDI exposure and subsequent follow-up for 4.5 years who developed isocyanate asthma with dermatitis during the exposure period (sIgE values are shown as solid white columns). After change in workplace and no exposure to isocyanates for the last 5 years, his lung function improved but he continued to exhibit MDI-specific IgE antibodies, but no specific IgG antibodies (shown as solid gray columns; note that all measured IgG values were below the reference value <3 mg/L); n.d. = not determined

Figure 4a shows serum samples for individual patients with presumed isocyanate asthma (for patient data see Tables 3, 4). We have observed here that improved IgE assay (in-vapor vs. in-solution) may enhance the diagnostic sensitivity for individual patients. Additionally, one patient (pat#1, Tables 3, 4) was followed over a period of 9.5 years (after first MDI-asthma diagnosis in our outpatient clinic). The patient, man, 27 year old, smoker, with obstructive ventilation disorder, recurrent wheeze and difficulty in breathing was working on a machine bending wood bands (spruce) with heated MDI containing glue for braces, post and bridges (the later were hand-notched, glued and doweled into ribs). He developed isocyanate asthma and suffered dermatitis, showed NSBHR and positive SIC reactions, was positive to common allergens in SPT and also showed an immediate-type MDI-SPT reaction, and his total IgE values was 261 kU/L. Asthma improved and dermatitis symptoms were not observed after he changed his job and had no further contact to isocyanates in the following check-up periods. The specific IgE data cover 4 years of MDI exposure and 5.5 years free from exposure (Fig. 4b). Interestingly, significant levels of sIgE antibodies persisted in this patient throughout the 4 years subsequent to the MDI exposure. This was a surprising result and contradicts the widely held belief that sIgE levels decay quickly upon the removal from exposure to isocyanate. Given the assumed short half-life of IgE (his specific IgG values were lower than 3 mg/L estimated non-specific reference values), this might be important for the diagnosis of patients currently no more exposed to isocyanates.

### Correlation with other diagnostic parameters and the antibody data

#### Presumed MDI-asthma cases (group A)

The specific IgE-/IgG-binding data were compared with other diagnostic parameter (see Tables 1, 2 for diagnostic parameter and supplementary Fig. 1 for the diagnostic flow chart). Interestingly, all patients with high specific IgE binding gave also a positive MDI-skin-prick test result. All patients in this group also exhibited a positive SIC response when challenged with MDI. In the patient group without MDI-sIgE antibodies, all but one had negative MDI-skin-prick results; NSBHR was both present and absent, the SIC results were positive and negative, and all had IgG antibodies at low levels. When looking closer at individual patients, the presumed MDI-asthma diagnosis could be confirmed by clinical findings, symptoms and cross-shift course of lung function or SIC in 7 out of 12 patients, although only 4 patients in this group had specific IgE antibodies. However, the combination of positive MDI-SIC, MDI-SPT and specific IgE antibodies correlated with asthma diagnosis (with RR of 5.7, *P* < 0.001, *n* = 12), whereas MDI-HSA-specific IgE alone showed RR of 1.28, *P* < 0.50 (when correlated with the clinical OA_I_ diagnosis) given the limitation of the small patient group. There was no significant correlation between the presence of IgG antibodies and asthma diagnosis (RR 0.4, *P* > 0.5). Interestingly, patients out of the IgE-negative group were diagnosed with MDI-induced hypersensitivity pneumonitis, with typical systemic and pulmonary symptoms and respective MDI-provoked SIC responses. The IgG binding (in combination with the positive SIC data) could be positively correlated (RR 1.2, *P* < 0.50) with the clinical diagnosis of P_I_.

#### Control groups (B, C, D)

Table 4 also provide data from a field study including a small group of 6 industrial workers with exposure to MDI (~5 ppb). The subjects were diagnosed directly in the workplace (only serum and urine samples were taken to the laboratory). None of the workers had asthmatic symptoms, as defined by the questionnaire, and had no evidence of airway obstruction, with all having FEV1 > 80 % predicted and FEV1/FVC higher than predicted-1 SD. However, 5 had work-related upper respiratory and conjunctival symptoms diagnosed in the course of examination. None of these MDI-exposed workers had significant detectable IgE or IgG antibodies. It has to be noted that both air monitoring and the spirometry were performed during the whole working week.

Table 5 shows control groups: 13 unexposed subjects and 12 patients with occupational asthma not exposed to isocyanates (baker’s asthma). None of the unexposed controls had MDI-specific IgE antibodies, one had sIgG binding at a low level (3.3 mg/L), and a similar result showed one control baker’s asthma patient.

**Table 5:** Demographic and clinical and functional characteristics of two control groups: healthy subjects (group c) and asthma patients, not exposed to isocyanates (group D, patients with baker’s asthma)

Subject	Demographic data			Immunological status		Lung function			MDI-specific antibodies		Final clinical diagnosis
No. #	Sex	Age	Smo-king status	Comm. allerg.	Total IgE kU/L	FVC % pred	FEV1 % pred.	NS-BHR	MDI-sIgE kU/L	MDI-sIgG mg/L	
Group C: Unexposed healthy control subjects											
19	F	28	No	Neg.	n.d.	n.d.	n.d.	n.d.	<002	<3	H
20	M	28	No	Pos.	n.d.	n.d.	n.d.	n.d.	<0.02	<3	H
21	F	50	No	Pos.	n.d.	n.d.	n.d.	n.d.	<0.02	3.3	H
22	F	54	No	Neg.	n.d.	n.d.	n.d.	n.d.	<0.02	<3	H
23	M	56	No	Neg.	n.d.	n.d.	n.d.	n.d.	<0.02	<3	H
24	M	30	No	Pos.	67	n.d.	n.d.	n.d.	<0.02	<3	H
25	F	31	No	Neg.	128	n.d.	n.d.	n.d.	<0.02	<3	H
26	M	55	Ex	Neg.	27	n.d.	n.d.	n.d.	<0.02	<3	H
27	F	57	No	Neg.	272	n.d.	n.d.	n.d.	<0.02	<3	H
28	F	61	No	Neg.	7.3	n.d.	n.d.	n.d.	<0.02	<3	H
29	F	47	No	Pos.	870	n.d.	n.d.	n.d.	<0.02	<3	H
30	F	43	Yes	Neg.	33	n.d.	n.d.	n.d.	<0.02	<3	H
31	M	40	No	Pos.	42	n.d.	n.d.	n.d.	<0.02	<3	H
Group D. Asthma patients not exposed to isocyanates											
32	M	42	No	Pos	83	88	86	neg.	<0.02	<3	OA_B_
33	M	40	No	Pos	135	94	92	Pos.	<0.02	<3	OA_B_
34	M	44	No	Pos	893	106	90	Pos.	<0.02	<3	OA_B_
35	F	62	Ex	Neg	65	115	105	Neg.	<0.02	<3	OA_B_
36	F	41	Yes	Pos	197	112	111	Pos.	<0.02	<3	OA_B_
37	M	57	Yes	Pos	246	95	80	Pos.	<0.02	<3	OA_B_
38	M	56	Ex	Neg	332	85	81	Neg.	<0.02	<3	OA_B_
39	M	50	Ex	Pos	33	83	66	Pos.	<0.02	<3	OA_B_
40	M	41	No	Pos	22	108	82	Neg.	<0.02	<3	OA_B_
41	M	45	No	Pos	101	102	98	Pos.	<0.02	<3	OA_B_
42	M	39	No	Pos	323	111	97	Neg.	<0.02	<3	OA_B_
43	M	50	No	Neg	153	107	75	Pos.	<0.02	4.86	OA_B_

See Table 1 for details, *OAB,* occupational baker’s asthma; *H,* healthy

## Discussion

### Are the antibody data valuable for the MDI-asthma diagnosis?

We could confirm our earlier studies (Baur 1983, 2007), showing the correlation between specific IgE antibodies and the diagnosis of isocyanate asthma using validated fluorescence immunoassay and detailed comprehensive clinical diagnosis. We did not observe false-positives, and the absence of specific IgE antibody is, however, not sufficient for excluding a diagnosis of isocyanate asthma. The presence of MDI-HSA-specific IgE antibodies was associated with immediate MDI-SPT responses and the clinical diagnosis of isocyanate asthma, although about half of MDI-asthmatic patients have no detectable specific IgE antibody. Also, (Tee et al. 1998) came to the conclusion that IgE is a specific, but insensitive index of occupational asthma. In a contrary, (Aul et al. 1999) suggested a primary role for IgG in various subjects with respiratory reactions to isocyanates. Also, others have documented IgG antibodies in patients with occupational asthma (Hur et al. 2008). Bernstein (Bernstein et al. 1993) recognized 3 MDI-asthma cases in 243 workers exposed to low MDI levels and detected both sIgG and sIgE binding to MDI-HSA in 2 out of 3 diagnosed isocyanate asthma cases (unfortunately, no original antibody levels were provided by the authors). There is a difference, however, between this study, in which currently exposed factory workers were screened and our study aiming to proof the diagnostic values of antibody testing for patients with already presumed asthma diagnosis. The most, analyzed collectives differ in the intensity of the symptoms, and the authors have applied in-solution conjugates, which appear to be at least 5-times less sensitive. The same group has analyzed later 9 exposed workers and 9 non-exposed control subjects and suggested that IgG might be a primary marker of isocyanate exposure rather than a diagnostic marker for isocyanate asthma (Lushniak et al. 1998). In our test group, two patients with diagnosed clinical asthma had elevated specific IgG antibodies in the absence of a specific IgE signal, one isocyanate asthma patient showed neither IgE nor IgG antibodies specific for MDI-HSA. (Vandenplas et al. 1993) described hypersensitivity pneumonitis-like responses in 2 out of 9 wood chip board workers applying MDI. The authors showed comprehensive diagnosis with detailed clinical parameter survey; unfortunately, they did not provide detailed information on the laboratory analysis precluding any data comparison. (Hur et al. 2008) analyzed 58 car upholstery workers currently exposed to MDI and reported 5 isocyanate asthma and 2 MDI-induced hypersensitivity pneumonitis cases. The authors measured sIgG antibodies in 8 and sIgE antibodies in 4 workers and showed further that the prevalence of specific IgG antibodies to MDI-HSA conjugate was higher (20.7 %) than for sIgE antibodies (8.6 %). Again, the study was designed to screen currently exposed subjects in a field study.

We could not confirm that low sIgG levels may provide a good marker for the MDI exposure, since in our control group not only 1 out of 6, but also two control subjects (without isocyanate exposure) showed positive sIgG results. On the other hand, we cannot rule out that IgG might be an exposure marker; further studies with both well-characterized patients and assay methods are needed to draw firm conclusions.

### Immunological analysis

We have observed here that improved IgE assay may enhance the diagnostic sensitivity for individual patients. High IgE binding using in-vapor HDI and TDI conjugates has been shown by others (Wisnewski 2007; Campo et al. 2007) and we have confirmed this for MDI as well, providing here additional information on commercially available MDI-CAP method. We could record some false-positive IgG values in the low range using commercial Phadia assay. In contrast, high levels of specific IgG antibodies were only associated with hypersensitivity pneumonitis (MDI alveolitis) in all assays. Recently, another group has characterized HSA-MDI conjugates prepared in-solution with a liquid MDI form and has shown specific IgG binding for 14 MDI-HSA-reaction sites (Wisnewski et al. 2010). Since there appears to be no association between IgG binding and MDI asthma (Lushniak et al. 1998), it would be interesting to test whether the IgG-specific structures are also related to specific IgE sites. Data from other groups (Kumar et al. 2009; Wisnewski 2007) indicate significant changes in the shape and charge of human albumin after exposure to HDI or MDI in humans or rats. Sabbioni and his group were the first to characterize the MDI-lysine adducts of albumin formed in vivo in detail and they found MDI-Lys and AcMD-Lys in the serum of MDI-exposed workers from construction sites and factories (Sabbioni et al. 2010). While this is a big step forward, it is not yet known whether the formation of these human MDI-albumin-adducts correlates with specific antibody responses. Further studies using characterized HSA-isocyanate conjugates in validated immunological tests and well-defined patient collectives are needed.

In order to better compare between the studies, the methods for the immunological analysis of the IgE and IgG antibodies need standardization and validation. Semi-automatic ImmunoCAP analysis could be the method of choice, since the RAST methodology (Spiazzi et al. 1991) is not available any more. It has also to be noted that the practical clinicians have rarely access to research centers using their own characterized conjugates for antibody testing and have to relay rather on the routine laboratories (using commercially available tests). It is important to test the validity of such tests and the art of the data interpretation. Only a few studies at all (using either HDI, or TDI conjugates) have compared different assay methods in-solution or in-vapor (Wisnewski 2007; Wisnewski et al. 2004); no recent study has made any attempts to compare the antibody data drawn with the commercial assays, most of the occupational and environmental practitioners relay on.

Additionally, we could not find any association with the amounts of the total IgE or with the atopy status in this study but it cannot be excluded that the low total IgE status (as seen in some patients) might reflect a low capability of producing specific antibodies.

### Non-IgE-driven pathomechanisms

It remains also to be clarified how many cases involve non-IgE pathomechanisms. Analyzing 13 isocyanate asthma patients (5 with positive and 7 with negative SIC results), Jones et al*.* (Jones et al. 2006) proposed a non-IgE-mediated (with Il-5-, CD25- and CD4) mechanism for isocyanate asthma, with a reservation that presumably this assumption might concern the IgE-non-responding group only. Also, matrix metalloproteinase-9 (MMP-9), ferritin, and transferrin (Palikhe et al. 2011 and monocyte chemotractant protein-1 (MCP-1) (Bernstein et al. 2002) were proposed. Further studies are necessary.

### Comprehensive clinical diagnosis is necessary

The diagnosis isocyanate asthma is known to be difficult as its patterns might be associated with isolated late asthmatic reaction, a biphasic dual reaction or an atypical reaction (Tarlo et al. 2008; Curwick et al. 2006; Hendrick 2002). Diagnosis of isocyanate asthma may be also difficult due to concurrent inflammatory rhinoconjunctivitis or COPD, leading to false-positive as well as false-negative diagnoses. Careful utilization of several diagnostic parameters is required for the evaluation of data. (Curwick et al. 2006; Hendrick 2002). Frequently, analyses of reported clinical cases relay simply on the opinions of individuals, and reliance on publications is further compromised by the frequency of misdiagnosis of occupational asthma. Though the positive SIC result is considered as a “gold standard” for isocyanate asthma, the comprehensive clinical asthma diagnosis is far more than SIC only. We found that all SIC-positive patients with sIgE antibodies and the MDI-asthma diagnosis have also shown positive MDI-SPT reaction, whereas SIC-positive hypersensitivity pneumonitis patients were negative for MDI-SPT response. Since SIC can only be performed in a few highly specialized centers, this result might be interesting for those having no access to this diagnostic test. The attributable proportion of occupational agents to the total asthma burden is in the range of 5–25 %, with isocyanates as one of the most important causes worldwide, reinforcing the acute need for a reliable diagnostic tests (Hendrick 2002).

## Conclusions

The isocyanate-specific IgE antibodies are not always detectable but their presence can be predictive of isocyanate asthma and supportive for the diagnosis of occupational asthma. In contrast, the presence of IgG antibody only appears to be indicative in hypersensitivity pneumonitis and not relevant in cases of isocyanate asthma. The MDI-specific prick test may provide additional supportive information, allowing differentiation between isocyanate asthma and MDI-provoked hypersensitivity pneumonitis. Thus, a carefully evaluated clinical diagnosis is paramount in each individual case.

## Electronic supplementary material

Below is the link to the electronic supplementary material.

**Fig. 1 Isocyanat asthma diagnostic flow chart**. *see main text for details on facultative diagnostics (PDF 32.4 kb)

## Abbreviations

HSA: Human serum albumin
MDI: 4,4′ Methylenediphenyl diisocyanates or diphenylmethane 4,4′-diisocyanates or 1-isocyanate-4 [4-isocyanatephenyl) methyl] benzene
OA_I_: Occupational isocanate asthma
P_I_: Isocynate induced hypersensitivity pneumonitis
