# Supplementary figures and images for: Is specific IgE antibody analysis feasible for the diagnosis of methylenediphenyl diisocyanate-induced occupational asthma?
Source: Int Arch Occup Environ Health. 2012 Apr 28;86(4):417–30. doi: 10.1007/s00420-012-0772-6 (PMC3633778; doi:10.1007/s00420-012-0772-6)

## Isocyanat asthma diagnostic flow chart

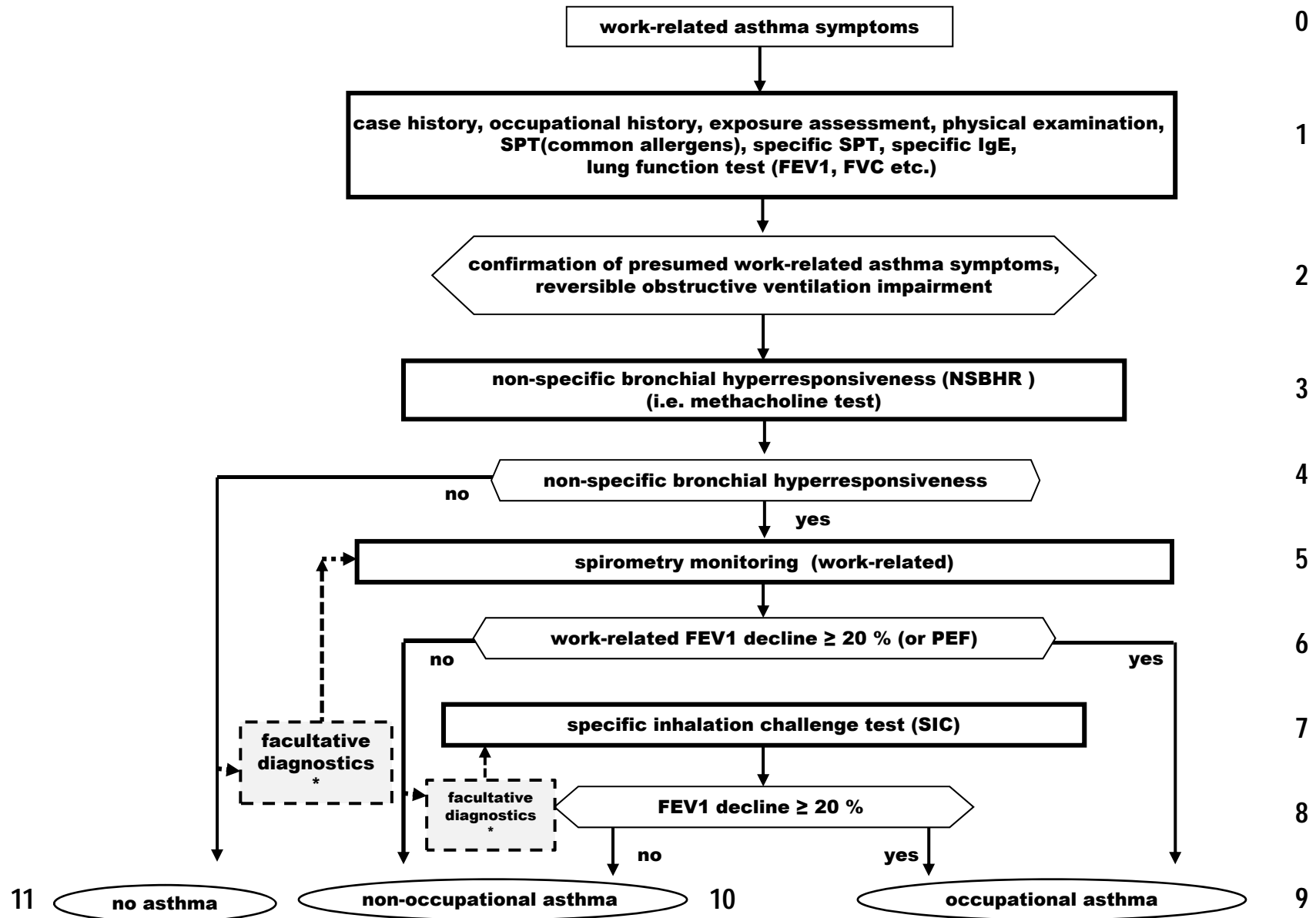

Supplement: Supplementary file 1 — Fig. 1 Isocyanat asthma diagnostic flow chart. *see main text for details on facultative diagnostics (PDF 32.4 kb) [file 420_2012_772_MOESM1_ESM.pdf]
